# Risk Stratification in Idiopathic Dilated Cardiomyopathy Patients Using Cardiovascular Coupling Analysis

**DOI:** 10.3389/fphys.2019.00841

**Published:** 2019-07-09

**Authors:** Javier Rodriguez, Steffen Schulz, Beatriz F. Giraldo, Andreas Voss

**Affiliations:** ^1^Institute for Bioengineering of Catalonia, The Barcelona Institute of Science and Technology, Barcelona, Spain; ^2^Automatic Control Department (ESAII), Barcelona East School of Engineering (EEBE), Universitat Politècnica de Catalunya, Barcelona, Spain; ^3^Institute of Innovative Health Technologies, Ernst-Abbe-Hochschule Jena, Jena, Germany; ^4^Centro de Investigación Biomédica en Red de Bioengenieria, Biomateriales y Nanomedicina, Madrid, Spain

**Keywords:** idiopathic dilated cardiomyopathy, heart rate variability, blood pressure variability, coupling analysis, sudden cardiac death, risk stratification

## Abstract

Cardiovascular diseases are one of the most common causes of death; however, the early detection of patients at high risk of sudden cardiac death (SCD) remains an issue. The aim of this study was to analyze the cardio-vascular couplings based on heart rate variability (HRV) and blood pressure variability (BPV) analyses in order to introduce new indices for noninvasive risk stratification in idiopathic dilated cardiomyopathy patients (IDC). High-resolution electrocardiogram (ECG) and continuous noninvasive blood pressure (BP) signals were recorded in 91 IDC patients and 49 healthy subjects (CON). The patients were stratified by their SCD risk as high risk (IDC_HR_) when after two years the subject either died or suffered life-threatening complications, and as low risk (IDC_LR_) when the subject remained stable during this period. Values were extracted from ECG and BP signals, the beat-to-beat interval, and systolic and diastolic blood pressure, and analyzed using the segmented Poincaré plot analysis (SPPA), the high-resolution joint symbolic dynamics (HRJSD) and the normalized short time partial directed coherence methods. Support vector machine (SVM) models were built to classify these patients according to SCD risk. IDC_HR_ patients presented lowered HRV and increased BPV compared to both IDC_LR_ patients and the control subjects, suggesting a decrease in their vagal activity and a compensation of sympathetic activity. Both, the cardio -systolic and -diastolic coupling strength was stronger in high-risk patients when comparing with low-risk patients. The cardio-systolic coupling analysis revealed that the systolic influence on heart rate gets weaker as the risk increases. The SVM IDC_LR_ vs. IDC_HR_ model achieved 98.9% accuracy with an area under the curve (AUC) of 0.96. The IDC and the CON groups obtained 93.6% and 0.94 accuracy and AUC, respectively. To simulate a circumstance in which the original status of the subject is unknown, a cascade model was built fusing the aforementioned models, and achieved 94.4% accuracy. In conclusion, this study introduced a novel method for SCD risk stratification for IDC patients based on new indices from coupling analysis and non-linear HRV and BPV. We have uncovered some of the complex interactions within the autonomic regulation in this type of patient.

## Introduction

According to the 2015 update of the heart disease and stroke statistics of the American Heart Association (Mozaffarian et al., 2015), ~325,000 cases of sudden cardiac death (SCD) occurred in the United States in that year, and it is the cause of 15–20% of mortality worldwide (Saour et al., 2017). The implantable cardioverter defibrillator (ICD) is commonly recommended in patients that are at high risk of suffering SCD, and the risk of SCD is halved when one is implanted, although the presence or absence of an ICD implant has no significant influence over the rate of death itself (Kober et al., 2016).

The implantation of an ICD is suggested in patients with an ejection fraction (EF) lower or equal to 35%. Currently, there is no effective means to stratify SCD risk in patients with EF above the risk threshold, who constitutes at least 70% of the patients who will suffer SCD (Chugh, 2017). Additionally, the effectiveness of ICD therapy is time dependent, making a prediction of the duration of the treatment desirable for the purpose of optimizing costs. Therefore, there is still a need for additional predictors to identify patients with idiopathic dilated cardiomyopathy (IDC) who have an increased risk of SCD and who benefit from ICD implantation (Bristow et al., 2004; Duray et al., 2006).

Previous studies have hypothesized various strategies to assess SCD risk based on clinical tests and data, imaging techniques, and signal processing methods, among others. The corrected QT interval was tested in elderly subjects and was associated with SCD risk (Panikkath et al., 2011); others analyzed T-wave inversions, wide QRS-T angle, and the left bundle branch block and found it prolonged the ability of QRS to predict all-cause mortality, including SCD (Aro et al., 2011, 2012a,b), but despite being useful for prediction, it is unable to predict individual risk. Sympathetic dominance of the autonomic nervous system in conjunction with pro-arrhythmic processes increases the probability of SCD in patients with ventricular fibrillation problems (Schwartz et al., 1992).

Studies related to cardiac imaging have analyzed the myocardial scar, including the peri-infarction border zone, to stratify SCD risk. The quantification of the total myocardial scar was explored, proving to be superior to the left ventricular ejection fraction (LVEF) in predicting an appropriate ICD therapy in cardiomyopathy patients (Bertini et al., 2012). Other studies demonstrated that the image-based analysis of myocardial scars can contribute to the decision to implant ICDs in SCD patients (Schmidt et al., 2007; Roes et al., 2009; Wu et al., 2012).

Heart rate variability (HRV) has been studied as a measurement of autonomic tone. Higher vagal tone activity is related to increased spontaneous variations in heart rate, and multiple non-linear techniques have been applied to study it (Wolf et al., 1978; Task-Force-of-the-European-Society-of-Cardiology-the-North-American-Society-of-Pacing-Electrophysiology, 1996; Ebrahimzadeh et al., 2014; Wu et al., 2014; Fujita et al., 2016). Lower levels of indices related to this variability have been associated with patients at SCD risk, regardless of their LVEF (La Rovere et al., 1998, 2003). Another index explored is heart rate turbulence, a consistent phenomenon in low-risk ischemic heart disease patients, as a measure of autonomic function. It is capable of predicting SCD-related mortality by assessing the absence of this behavior (Schmidt et al., 1999). The dynamics of the cardiovascular system behave in a highly complex way through the interplays of different linear and non-linear subsystems (Voss et al., 2009). Changes in blood pressure are reflected in changes in heart-rate regulation, and vice versa (Cohen and Taylor, 2002).

Several linear and non-linear time series analysis approaches have been developed for the quantitative analysis of the cardiovascular system in bivariate ways. However, linear approaches might be insufficient to quantify non-linear structures and the complexity of physiological systems. Therefore, approaches from non-linear time-series analysis seem to be more suited to capture complex interactions between time series, and are able to quantify direct interrelationships such as the nonlinear influence of blood pressure on heart rate. These coupling approaches are used to quantify direct and indirect relationships, as well as causal and non-causal relationships between time series, providing deeper insights into alterations of the cardiovascular system and leading to improved knowledge of the interacting regulatory mechanisms under different physiological and pathophysiological conditions. These approaches represent promising tools for detecting multivariate information flows (Schulz et al., 2013a). Some studies are based on the analysis of these interactions through the application of bivariate coupling methods.

For example, the directional cardiovascular interactions on young healthy subjects were assessed by bivariate and multivariate coupling measures, finding that bivariate measures better quantify the information transferred between indices, while trivariate better reflects the existence and delay of directed interactions (Javorka et al., 2017). Information decomposition measurements, in terms of variance or entropy, were explored to assess information dynamics in cardiovascular networks (Faes et al., 2017), analyzing the heart period, the systolic blood pressure, and the respiratory activity. The authors concluded that these measures of information transfer and information modification are better assessed through entropy-based and variance-based methods. Other work explored the polysomnographic recordings and the finger blood pressure measurements in healthy subjects in order to investigate the differences between the wake-sleep states in the heart period and the systolic blood pressure coupling (Silvani et al., 2008). They found that at low frequencies there are differences between these states in human subjects. Additionally, the complexity and causality of the interactions of cardiovascular variability series was assessed through linear model-based and non-linear model free techniques (Porta et al., 2014), and deducing that model free methods provide additional insights compared to the simpler linear model based approaches.

The behavior of cardiovascular coupling differs based on physiological conditions. Consequently, we hypothesize that the relationships between the cardiac and vascular systems will be different between the IDC patients at a high and low risk of SCD. Therefore, the aim of this study is to analyze the suitability of cardiovascular couplings for risk stratification in these patients. We propose the characterization of these interactions through features extracted from ECG and blood pressure signals, to better describe the complex dynamics of the cardiovascular interaction and identify new indices of cardiovascular risk.

## Database

The signals analyzed in this study are part of German Autonomic Regulation Trial (ART) study, oriented to evaluate risk predictors of SCD and to improve the risk stratification in IDC patients. Signals from 220 IDC patients were recorded in two hospitals: the Friedrich-Schiller University Hospital in Jena (44 patients) and the Franz-Volhard Clinic in Berlin (176 patients). All participants provided their written informed consent to a protocol approved by the local ethics committee of the two hospitals. This study complies with the Declaration of Helsinki. The study was approved by the local Ethics Committee (No. 0986-11/02).

In the acquisition protocol, the ECG signals (32 bit resolution, 1,600 Hz sampling frequency) and blood pressure (32 bit, 500 Hz) were synchronously recorded for 30 min. The ECG recordings were performed with a Porti system (TMSi BV, Netherlands), and the blood pressure recordings with the Portapress NIBP monitor (TNO Biomedical Instrumentation, Amsterdam, the Netherlands). All patients were recorded under resting conditions in supine position.

The inclusion criteria for the IDC patients were LVEF <45% and/or fractional shortening <25%, and the left ventricle end-diastolic diameter (LVEDD) >117%. Additionally, the New York heart association index (NYHA) of each patient was included. The exclusion criteria were systemic hypertension at rest, coronary artery disease, congenital heart disease, pericardial diseases, valvular heart diseases, systemic disease known to cause dilated cardiomyopathy, chronic alcoholism, sustained ventricular tachycardia, atrial fibrillation, diabetes mellitus, renal failure, and active permanent cardiac pacemaker as well as patients without sinus rhythm (Voss et al., 2010, 2012b).

Afterwards, an additional exclusion criteria was applied to discard patients with comorbidities and confounding factors influencing the autonomic regulation system. 129 of the 220 patients were excluded due to the following reasons: 36 with paced rhythm, 34 patients suffered coronary artery disease, 32 presented a high (>5%) percentage of ectopic beats or artifacts, 19 with atrial fibrillation, four because of technical problems, two suffered from hypertrophic non-obstructive cardiomyopathy, one patient with arrhythmogenic right ventricular cardiomyopathy, and another patient who was clinically unstable due to acute decomposition (Voss et al., 2012b). Finally, a total number of 91 IDC patients (21 female, 70 male) were investigated in this work.

After a median follow-up period of 28 months (range: 17–38 months), the patients were classified into two groups according to their SCD risk. The group of patients that remained in stable physical condition were considered as low risk for cardiovascular sudden death (*IDC*_*LR*_). The remaining patients, who either died of SCD or needed resuscitation because of a life-threatening tachyarrhythmia, were categorized as high risk for cardiac sudden death (*IDC*_*HR*_). None of these patients died from a non-cardiac disease. Additionally, 49 healthy subjects (30 male, 19 female; aged 46 ± 14 years) were used as control group (CON). Table 1 presents the baseline clinical information of the IDC patients.

**Table 1:** Baseline clinical information of ART database IDC patients (median and interquartile range).

	**IDC_**HR**_** ***N* = 77 (59 ♂, 18 ♀)**	**IDC_**LR**_** ***N* = 14 (11 ♂, 3 ♀)**	***p*-value**
Follow-up duration [months]	27 [17; 37]	30 [21; 38]	n.s.
Age (years)	55 [50; 60]	56 [50; 63]	n.s.
LVEF (%)	29 [27; 37]	35 [27; 46]	n.s.
LVEDD (mm)	69 [61; 79]	61 [58; 68]	n.s.
LVESD (mm)	60 [53; 69]	49 [44; 56]	0.0028
NYHA	3 [2; 4]	2 [2; 3]	0.0024

*IDC_HR_ and IDC_LR_, high risk and low risk group of patients with dilated cardiomyopathy; N, number of patients within the groups (♂ = Male; ♀ = Female); LVEF, Left ventricular ejection fraction; LVEDD, Left ventricular end-diastolic diameter; LVESD, left ventricular end-systolic diameter; NYHA, New York heart association index*.

In order to characterize autonomous regulation of the cardiovascular systems, the following time series from ECG (RR interval) and BP signals were extracted using in-house software (programming environment Delphi 3 and MATLAB® R2011b):

- Time series of heart rate consisting of successive beat-to-beat intervals [BBI, tachogram, (ms)] from the ECG signal.
- Time series consisting of the maximum successive end-systolic blood pressure amplitude values over time in relation to the previous R-peak [SBP, systogram, (mmHg)] from the BP signal.
- Time series consisting of the minimum successive end-diastolic blood pressure amplitude values over time in relation to the previous R-peak [DBP, diastogram, (mmHg)] from the BP signal.

All extracted time series (BBI, SBP, DBP) were filtered by applying an adaptive variance estimation algorithm (Wessel et al., 2000) to remove and interpolate seldom occurring ventricular premature beats and artifacts (e.g., movement, electrode noise, and extraordinary peaks) to obtain normal-to-normal beat time series (NN). To obtain synchronized time series, BBI, SBP, and DBP were resampled using a linear interpolation method (2 Hz).

## Methods

The BBI, SBP, and DBP time series were extracted using algorithms based on zero crossings and different thresholds. These data were evaluated through several non-linear characterization techniques such as high resolution joint symbolic dynamics (HRSJD), segmented Poincaré plot analysis (SPPA) and normalized short-time partial directed coherence (NSTPDC).

### High Resolution Joint Symbolic Dynamics

The joint symbolic dynamics (JSD) method (Baumert et al., 2002) is based on the analysis of dynamic processes by the means of symbols. Considering BBI, SBP, and DBP time series, ***X***is defined as a bivariate sample vector that contains two out of the three time series, for all their possible combinations (BBI-SBP, BBI-DBP, and SBP-DBP), as expressed in Equation (1),

(1) $$\begin{array}{c} X^{BBI\_SBP}={\left[x_{n}^{BBI},\;x_{n}^{SBP}\right]}^{T\;}\;\\ \;X^{BBI\_DBP}={\left[x_{n}^{BBI},\;x_{n}^{DBP}\right]}^{T}\;\\ \;X^{SBP\_DBP}={\left[x_{n}^{SBP},\;x_{n}^{DBP}\right]}^{T}\; \end{array}\}n=0,1,\ldots ,\;N\;with\;x\in \mathbb{R},$$

being *N* the total number of samples.

In JSD, the increments between two successive values of the temporal series are coded as “1” and the decrements and equilibriums are coded as “0,” these increments and decrements are considered in relation to a threshold *l*. The vector ***X***can be transformed into the symbolic vector ***S***using the rules set out in Equation (2), were the threshold *l* is set equal to 0 (Baumert et al., 2002; Giraldo et al., 2015).

(2) $$\begin{array}{l} \begin{array}{c} S^{BBI\_SBP}={\left[S_{n}^{BBI},\;S_{n}^{SBP}\right]}^{T}\\ S^{BBI\_DBP}={\left[S_{n}^{BBI},\;S_{n}^{DBP}\right]}^{T}\\ \;S^{SBP\_DBP}={\left[S_{n}^{SBP},\;S_{n}^{DBP}\right]}^{T}\; \end{array}\}\;n=0,1,\ldots ,\;N,\;with\;S\in 0,1\\ \;\;S_{n}^{BBI}=\left\{ \begin{array}{l} 0:\left(x_{n+1}^{BBI}-x_{n}^{BBI}\right)\;\leq \;0\\ 1:\left(x_{n+1}^{BBI}-x_{n}^{BBI}\right)\;>\;0 \end{array}\right.\\ \;S_{n}^{SBP}=\left\{ \begin{array}{l} 0:\left(x_{n+1}^{SBP}-x_{n}^{SBP}\right)\;\leq \;0\\ 1:\left(x_{n+1}^{SBP}-x_{n}^{SBP}\right)\;>\;0 \end{array}\right.\\ \;S_{n}^{DBP}=\left\{ \begin{array}{l} 0:\left(x_{n+1}^{DBP}-x_{n}^{DBP}\right)\;\leq \;0\\ 1:\left(x_{n+1}^{DBP}-x_{n}^{DBP}\right)\;>\;0 \end{array}\right. \end{array}$$

Sequences of symbols are considered words of length *k*. The words are then arranged in a vector matrix ***W***. Particularly, for *k* = 3 (*S*_*n*_, *S*_*n*+1_, *S*_*n*+2_) an 8 ×8 vector matrix can be derived, taking values from (000, 000)^*T*^ to (111, 111)^*T*^.

In order to obtain JSD indices that are more robust against noise, fluctuations and artifacts, the comparison threshold should be something other than 0. There are several advantages for choosing a non-zero threshold. For instance, a state will be generated that will help to distinguish between small and large changes in the system's variability. It is also possible to differentiate between decrements and equilibrium because both states are no longer coded with the same symbols. Lastly, the number of word types including “0” will not be the most prevalent within the ***W***matrix (Schulz et al., 2013b, 2015b). The high-resolution joint symbolic dynamics (HRJSD) method implements a three symbol JSD after setting a threshold *l*. The increment states are coded as “2,” the decrements are coded as “1” and the equilibrium states are coded as “0.” With this technique, the transformation from ***X***to ***S***varies as is shown in Equation (3).

(3) $$\begin{array}{l} S_{n}^{BBI}=\left\{ \begin{array}{c} 0:\left(x_{n+1}^{BBI}-x_{n}^{BBI}\right)<-l^{BBI}\\ 1:-l^{BBI}\leq \left(x_{n+1}^{BBI}-x_{n}^{BBI}\right)\leq \;l^{BBI}\\ 2:\left(x_{n+1}^{BBI}-x_{n}^{BBI}\right)>\;l^{BBI} \end{array}\right.\\ S_{n}^{SBP}=\left\{ \begin{array}{c} 0:\left(x_{n+1}^{SBP}-x_{n}^{SBP}\right)<-l^{SBP}\\ 1:-l^{SBP}\leq \left(x_{n+1}^{SBP}-x_{n}^{SBP}\right)\leq \;l^{SBP}\\ 2:\left(x_{n+1}^{SBP}-x_{n}^{SBP}\right)>\;l^{SBP} \end{array}\right.\\ S_{n}^{DBP}=\left\{ \begin{array}{c} 0:\left(x_{n+1}^{DBP}-x_{n}^{DBP}\right)<-l^{DBP}\\ 1:-l^{DBP}\leq \left(x_{n+1}^{DBP}-x_{n}^{DBP}\right)\leq \;l^{DBP}\\ 2:\left(x_{n+1}^{DBP}-x_{n}^{DBP}\right)>\;l^{DBP} \end{array}\right. \end{array}$$

The new space is composed of combinations of 27 different possible types of words (from 000 to 222) and a total of 729 indices. All the word types were grouped into eight pattern families, transforming the vector matrix ***W***into a vector matrix family (***W***_***f***_**)**. These indices were analyzed by their occurrence probabilities. Afterwards, these indices were grouped according to their family description (Table 2). These pattern families represent different interactions between the branches of the autonomic regulation system, leading to indices with statistically sufficient occurrence probabilities (Schulz et al., 2015b). Additionally, the Shannon entropy (Rundle et al., 2019) was calculated for all the proposed families to assess the complexity of the coupling.

**Table 2:** Description of pattern families explored in the HRJSD method.

**Family**	**Description**
E0	No variation of 3 successive “0” symbols (“000”)
E1	No variation of 3 successive “1” symbols (“111”)
E2	No variation of 3 successive “2” symbols (“222”)
LU1	Low increasing behavior (“122,” “022,” “112,” “221,” “220,” “211,” “121,” “212”)
LD1	Low decreasing behavior (“011,” “001,” “002,” “110,” “100,” “200,” “010,” “101”)
LA1	Fast alternant behavior (“020,” “202”)
P	Alternant peak-like behavior (“120,” “201,” “210”)
V	Alternant valley-like behavior (“021,” “102,” “012”)

The thresholds applied were 5 ms and 1 mmHg on the BBI and the blood pressure time series, respectively. These threshold values were successfully applied in earlier work (Schulz et al., 2013b). The threshold level using spontaneous baroreflex sensitivity, in contrast to other thresholds, is the most suitable for highlighting different specific cardiovascular coupling patterns.

### Segmented Poincaré Plot Analysis

The Poincaré plot analysis (PPA) is used to quantify self-similarity in processes by plotting the data into a higher dimensional state space. Considering a time series *X*(*n*) = *x*_1_, *x*_2_, *x*_3_, …, *x*_*n*_, the Poincaré plot is obtained by plotting *X*(*n*) vs. *X*(*n*+1). The typical representation is an elongated scatter of plots through the line of identity with all the points whose values are near the mean placed toward the center. Long- and short-term variability indices can be by fitting an ellipse to the shape of the plot and measuring the dispersion along the minor (SD1) and major (SD2) axis of the ellipse, corresponding to their standard deviation (Rodriguez et al., 2017).

Although PPA is a non-linear characterization method, indices SD1 and SD2 can be correlated with the linear behavior of the system, hence making them a suboptimal way to explore information about the non-linear part of the process (Seeck et al., 2011; Voss et al., 2012a).

The Segmented Poincaré plot analysis (SPPA) is an enhanced pseudo-phase space quantification method that yields indices that also represent the non-linear information of the system. In SPPA the SD1 and SD2 indices are calculated similarly to PPA. Then, the scatter points are rotated α degrees around the main focus of the plot as defined in Equation (4),

(4) $$\left[\begin{array}{c} {X^{\prime }}_{n}\\ {X^{\prime }}_{n+1}\\ z^{\prime } \end{array}\right]=\left[\begin{array}{c} \bar{X_{n}}\\ \bar{X_{n+1}}\\ z \end{array}\right]+\left(\left[\begin{array}{ccc} \cos\alpha & -sin\alpha & 0\\ sin\;\alpha & \text{cos}\alpha & 0\\ 0 & 0 & 1 \end{array}\right]\times \left[\begin{array}{c} X_{n}-\bar{X_{n}}\\ X_{n+1}-\bar{X_{n+1}}\\ z \end{array}\right]\right).$$

The *x* and *y* axis correspond to *X*(*t*) and *X*(*t* + 1) values, $\bar{X_{n}}\;$and $\;\bar{X_{n+1}}$ are the mean values of the original and shifted ***X***time series respectively, and *z* is the axis of the rotation (Voss et al., 2010, 2012a; Seeck et al., 2011). In this case we define α = 45 degrees in order to simplify the estimating procedure of the SD1/SD2 adapted probability. Afterwards, a 12 ×12 rectangular grid is drawn for the plot. The size of the rectangles (height, width) is adapted based on the SD1 (row) and SD2 (column) values.

Finally, for each rectangle in position (*i, j*) the single probability (ρ_*ij*_) is calculated considering the number of points contained by the total number of points in the series. Afterwards, the probabilities of each row (ρ_*ri*_) and column (ρ_*cj*_) are calculated as the sum of their single probabilities, as shown in Equation (5):

(5) $$\begin{array}{l} \rho_{ri}=\sum_{j=\;1}^{12}\rho_{ij}\\ \rho_{cj}=\sum_{i=1}^{12}\rho_{ij}. \end{array}$$

### Normalized Short-Time Partial Directed Coherence

The directed coherence method (DC) describes how and whether two complex physiological signals are functionally connected (Faes et al., 2012). The DC method studies the relative structural relationships between the systems by breaking down their interactions into *feedback* and *feedforward* aspects.

The partial directed coherence method (PDC) determines the either direct or indirect causality between the systems analyzed. The PDC is limited to work on stationary signals and is unable to yield information about the partial correlative short-time interaction properties (Baccala and Sameshima, 2001).

The NSTPDC is able to manage non-stationary signals by evaluating their dynamic coupling changes and detecting their level and directions in multivariate and complex dynamic systems (Adochiei et al., 2013; Schulz et al., 2015a).

Normalized short-time partial directed coherence based on an *m*-dimensional multichannel auto-regressive model (MAR) process with model order *p* to determine Granger causality in the frequency domain. For the selection of the optimal model order p_*opt*_ of the AR(*p*) model and for the estimation of its coefficients, the stepwise least squares algorithm (Neumaier and Schneider, 2001) and the Schwarz's Bayesian Criterion (SBC) were applied (Schneider and Neumaier, 2001). NSTPDC is based on the time-variant partial directed coherence approach (tvPDC, π_*xy*_(*f, n*)) providing information about the partial correlative short-time interaction properties of non-stationary signals, with *f* as the frequency and *n* the number of windows (Milde et al., 2011).

To quantify the coupling direction between two time series, ***X***and ***Y***(e.g., BBI and SBP: with *x*_BBI_ and *y*_SBP_) with the covariate *z* (e.g., DBP with *z*_DBP_), a coupling factor (CF) was introduced. CF was obtained by dividing the mean value π_*xy*_(*f, n*) by the mean value of π_*yx*_(*f, n*), defined as

(6) $$\begin{array}{r} CF=\frac{\frac{1}{n}\sum \pi_{x_{BBI}y_{SBP}}\left(f,n\right)}{\frac{1}{n}\sum \pi_{y_{SBP}x_{BBI}}\left(f,n\right)},\bar{a}=\frac{1}{n}\sum \pi_{x_{BBI}y_{SBP}}\left(f,n\right),\\ \bar{\;b}=\frac{1}{n}\;\sum \pi_{y_{SBP}x_{BBI}}\left(f,n\right).\; \end{array}$$

These results were normalized to become a specific set of values leading to the (normalized) factor NF representing the coupling direction, given by

(7) $$\begin{array}{l} NF=\left\{ \begin{array}{l} 2,\;if\left(\max=\bar{a}\&\frac{\bar{a}}{\bar{b}}>5\right)\\ 1,\;if\;\left(\max=\bar{a}\&2<\frac{\bar{a}}{\bar{b}}\leq 5\right)and\\ 0,\;if(\max=\bar{a}\&0\leq \frac{\bar{a}}{\bar{b}}\leq 2) \end{array}\right.\\ NF=\left\{ \begin{array}{l} -2,\;if\left(\max=\bar{b}\&\frac{\bar{b}}{\bar{a}}>5\right)\\ -1,\;if\;\left(\max=\bar{b}\&2<\frac{\bar{b}}{\bar{a}}\leq 5\right).\\ \;0,\;if(\max=\bar{b}\&0\leq \frac{\bar{b}}{\bar{a}}\leq 2) \end{array}\right. \end{array}$$

A normalization procedure was applied to CF leading to the normalized factor (NF). NF determinates the direction of the causal connections between the investigated time series (*x*_BBI_ and *y*_SBP_) as a function of frequency *f*. NF takes the following values: NF = {−2, −1, 0, 1, 2}. Strong unidirectional coupling is indicated if NF is −2 or 2 (where −2 denotes *y*_SBP_ as the driver), bidirectional coupling if NF = −1 or 1 (−1 denotes *y*_SBP_ as the driver), and an equal influence in both directions and/or no coupling if NF = 0 in respect to coupling strengths (if both area indices reveal equal values that are larger than zero an equal influence in both directions is present, on the other hand, if both area indices reveal equal values but are zero there is no coupling).

For determining the coupling strength between two time series, e.g., *x*_BBI_ and *y*_SBP_ with covariate (*z*_DBP_), the areas (A_BBI → SBP(DBP)_, A_SBP → BBI(DBP)_, [a.u.]) generated in space by CF were estimated in each window within the frequency band *f* = 0–2 Hz, and afterwards, averaged. A_BBI → SBP(DBP)_ and A_SBP → BBI(DBP)_ ranges between 0 to 1 [0, 1]. Hereby, 1 indicates that all causal influence originating from time series ***X***are directed toward (arrows: →) time series ***Y***.

In order to take advantage of the aspect of stationarity and scale-invariance for NSTPDC analyses, a normalization (zero mean and unit variance) of the time series BBI, SBP, and DBP was performed (Schulz et al., 2015a). Therefore, each sample *i* of the BBI-, SBP-, or DBP- time series **X** = {*x*_*i*_, *i* = 1, …*N*} and **Y** = {*y*_*i*_, *i* = 1, …*N*} with *N* as the maximal number of samples *i* (temporal index) was first normalized by subtracting the mean of $\bar{x}$ and, then divided by the standard deviation (std) of ***X***or ***Y***, respectively. The normalized time series x_*norm*_ and y_*norm*_ (zero mean and unit variance) were thus obtained as seen in (8)

(8) $$x_{norm}\left(i\right)=\frac{x\left(i\right)-\bar{x}}{std\left(x\right)}\;and\;y_{norm}\left(i\right)=\frac{y\left(i\right)-\bar{y}}{std\left(y\right)}.$$

### Dual Sequence Method

A widely spread method to investigate the spontaneous baroreflex sensitivity (BRS) is the sequence method (Bertinieri et al., 1988). The BRS was obtained scanning the SBP and BBI time series for sequences of three or more successive heart beats in which a progressive increase (or decrease) in SBP was followed (with a one-beat delay). The dual sequence method (DSM) was developed to improve the analysis of the baroreflex sensitivity (Malberg et al., 1999, 2002). A fluctuation is defined when there are variations greater than 1 mmHg (increasing or decreasing) in SBP and greater than 5 ms in BBI values. The highest slope of every sequence was taken for the linear regression. The slopes of the regression lines between the SBP and BBI sequences were taken as an index for local BRS [ms/mmHg] and calculated in each recording. The DSM is based on standard sequence methods, the enhancement lies in the analysis of two different kinds of BBI response: bradycardic (an increase in SBP causes an increase in BBI) and tachycardic fluctuations (a decrease in SBP causes a decrease in BBI), whereas only the bradycardic fluctuations represented the classical spontaneous baroreflex sensitivity (*bslope*). The analysis of the tachycardic fluctuations (*tslope*) provides additional information about autonomous cardiovascular regulation (Parati et al., 2000).

### Heart Rate and Blood Pressure Variability Standard Indices

The HRV and the blood pressure variability (BPV) were quantified using the standard indices from time and frequency domain. The following indices were calculated from the BBI, SBP, and DBP time series, with NN being the normal-to-normal intervals:

- The mean of BBI, SBP, and DBP time series (BBI_meanNN, SBP_meanNN and DBP_meanNN, respectively).
- The standard deviation of BBI, SBP, and DBP time series (BBI_sdNN, SBP_sdNN, DBP_sdNN).
- The proportion derived by dividing NN50 (number of pairs of adjacent NN intervals differing by more than 50 ms in the entire recording) by the total number of NN intervals (BBI_PNN50, SBP_PNN50, DBP_PNN50).
- The square root of the mean squared differences of BBI, SBP, and DBP time series (BBI_rmssd, SBP_rmssd, DBP_rmssd).
- The power of the low frequency components (0.04–0.15 Hz) of BBI, SBP, and DBP time series (BBI_LF, SBP_LF, DBP_LF).
- The power of the high frequency components (0.15–0.4 Hz) of BBI, SBP, and DBP time series (BBI_HF, SBP_HF, DBP_HF).
- The ratio between the low and high frequency power components of BBI, SBP, and DBP time series (BBI_LF/HF, SBP_LF/HF, DBP_LF/HF).

### Feature Extraction

A total number of 621 indices were extracted to analyze the interaction between the coupling across all signals (ECG and blood pressure signals). The “cd” label represents the cardiac and diastolic coupling, “cs” label indicates the cardiac and systolic coupling, and “ds” label the diastolic and systolic coupling.

The distribution of these indices is as follows: 264 indices from the HRJSD, 216 from the SPPA, 97 from the JSD, 12 from the PPA, 9 from the NSTPDC, 21 from the standard HRV and BPV indices, and 2 from the DSMs. Summary descriptions of the indices are shown in Table 3.

**Table 3:** Coupling indices extracted from high-resolution joint symbolic dynamics, segmented Poincaré plot analysis, and normalized short-time partial directed coherence.

**Index**	**Description**
HRJSDxy_Fx-Fy	Probability of occurrence of the Fx and Fy word families from the x–y coupling
HRJSDShxy	Shannon entropy of all the word families from the x-y coupling
HRJSDxy-Fx	Summation of the occurrences of Fx on all the families from the x-y coupling
SPPAxy_Row_n-m	Probability of occurrence of the Row n-m from the x-y coupling
SPPAxy_Column_n-m	Probability of occurrence of the Column n-m from the x-y coupling
JSDxy-n	Probability of occurrence of the word n from the x-y coupling
PPAxy_SD1	Short-time standard deviation from the x-y coupling
PPAxy_SD2	Long-time standard deviation from the x-y coupling
PPAxy_SD1/SD2	Short and long deviations ratio from the x-y coupling
NSTPDCxy_NF	Normalized coupling factor from the x-y coupling
NSTPDCxy_ Ax → y	Coupling strength of x-y from x to y
NSTPDCxy_ Ay → x	Coupling strength of x-y from y to x

*x and y represent the couplings cd, cs, and ds; F represents the word families: E0, E1, E2, LU1, LD1, LA1, P and V; n and m represent the number of row and column in the PPA based indices, respectively*.

## Statistical Analysis

In order to reduce dataset dimensionality, Mann Whitney non-parametric statistical test was used to determine the statistical significance of the indices obtained in the characterization process. The results were analyzed for different levels of significance, including the Bonferroni criterion, considering:

**Table:** 

*n.s.*	*ρ ≥ 0.01*		
*	*ρ ≤ 0.01 significant*		
**	*ρ ≤ 0.001 highly significant*		
***	*ρ ≤ 0.0000167 Bonferroni criterion*		
	**n* = 621 indices*		

Additionally, a correlation analysis was performed on the statistically significant indices. The ones with high correlation (ρ ≥ 0.7) and relative lower significance were discarded.

The leave-one-out cross-validation procedure was used to validate the results. The classification results are presented in terms of accuracy (Acc), sensitivity (Sn), specificity (Sp), and area under the curve (AUC).

## Classification Techniques

The objective of the support vector machines (SVM) method is to find a higher dimensional space where a classification problem can be more easily solved than in the original space. The vectors that defines the hyperplane are called support vectors. This technique allows separating groups of data that are not originally possible using linear classifiers (Giraldo et al., 2015).

Being ***X*** = {*x*_1_, …, *x*_*L*_}, *x* ∈ ℝ a given set of data vectors and ***Y*** = {*y*_1_, …, *y*_*L*_} their corresponding labels, the SVM function, defined as a linear discriminant function, known as hyperplane, is given can be defined by Equation (9),

(9) $$f\left(x\right)=wz+b=\sum_{i}^{L}\alpha_{i}y_{i}K\left(x_{i}y_{i}\right)+b,$$

where *w* is the normal vector to the hyperplane. The function *K*(*x*_*i*_*y*_*i*_) is the Kernel function that will shape the hyperplane and α_*i*_ and *b* define the efficiency of the classifier on the optimal values. In this study we evaluated the Gaussian, Laplace, and ANOVA kernels.

The Gaussian kernel is useful to model radially distributed data, and is defined in Equation (10), where σ is a penalization term,

(10) $$K\left(x,y\right)=e^{-\left(\frac{{\left\|x-y\right\|}^{2}}{2\sigma^{2}}\right)}.$$

Laplace kernel is a less σ influenced version of the Gaussian kernel, given by Equation (11)

(11) $$K\left(x,y\right)=e^{-\left(\frac{{\left\|x-y\right\|}^{2}}{2\sigma }\right)}.$$

The ANOVA kernel works well on multidimensional support vector regression models (Stitson et al., 1999) and is defined by Equation (12), where σ and *d* are the optimization indices of its function,

(12) $$K\left(x,y\right)=\sum_{k=1}^{n}e^{{\left(-\sigma {\left(x^{k}-y^{k}\right)}^{2}\right)}^{d}}.$$

The classification problem is solved by maximizing the margin while minimizing the training error. Using the Lagrange multipliers method, a dual formulation can be obtained (Cortes and Vapnik, 1995) (Equation 13),

(13) $$minP\left(w,b\right)=\;\frac{1}{2}{\left\|w_{m}z\right\|}^{2}+C\sum_{i}K_{1}\left[y_{i}f(x_{i})\right]$$

where *C* is a penalty parameter. Besides the scale of *C* having no direct meaning, as its value increases, the penalty assigned to errors is stronger, narrowing the decision boundary (Ben-Hur et al., 2008).

Each feature was scaled and normalized (zero mean and unit variance) in order to avoid scaling biases. For each iteration of features, the model was built by optimizing the value of *C* for each of the kernels considered, by iterating different values of σ and *d*. The indices that showed statistical differences and low correlation were used in pairs to build several SVM models. The accuracy of each model was then calculated and the one with the higher value was chosen as optimal for each type of kernel.

## Results

The calculated indices were used to analyze the cardiovascular coupling in 91 IDC patients and 49 healthy subjects. Four different comparisons were performed:

- The high risk IDC patients (*IDC*_*HR*_) vs. the low risk IDC patients(*IDC*_*LR*_).
- The *IDC* patients vs. the CON subjects.
- The high risk IDC patients (*IDC*_*HR*_) vs. the CON subjects.
- The low risk IDC patients (*IDC*_*LR*_) vs. the CON subjects.

### **IDC**_**HR**_ Patients vs. **IDC**_**LR**_ Patients Compared With CON Subjects

**Figure 1:**
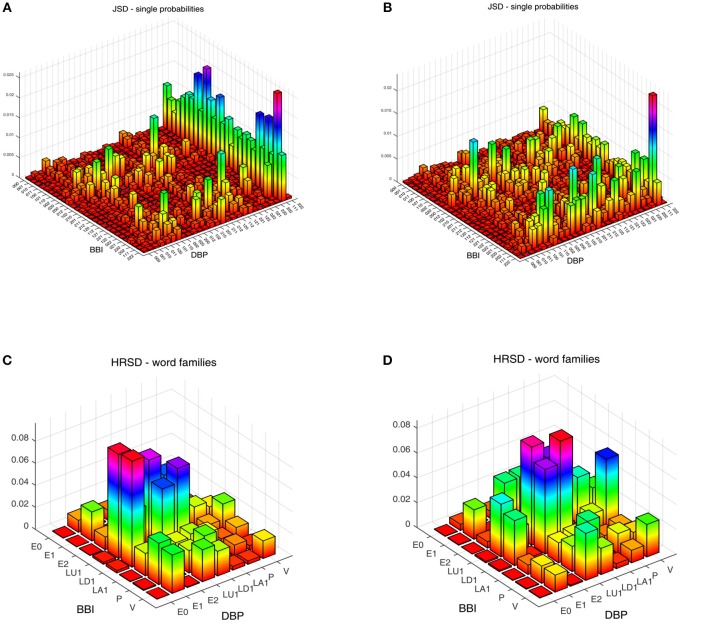
Three-dimensional plots of the word distribution density matrix using the HRJSD method (single word probabilities, word families), from a patient of **(A,C)** *IDC*_*LR*_ and **(B,D)** *IDC*_*HR*_, respectively, for cardio-diastolic coupling.

**Figure 2:**
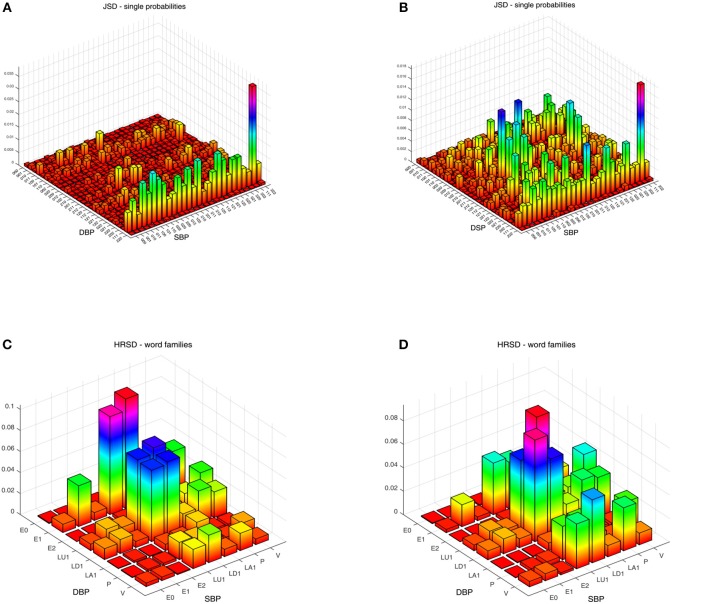
Three-dimensional plots of the word distribution density matrix using the HRJSD method (single word probabilities, word families), from a patient of **(A,C)** *IDC*_*LR*_ and **(B,D)** *IDC*_*HR*_, respectively, for diastolic-systolic coupling.

We obtained statistically significant differences in the symbolic dynamic analysis, for both the cardio-diastolic (BBI–DBP) and diastolic-systolic (DBP-SBP) couplings. Figures 1, 2 present an example of the three-dimensional plots of the word distribution density matrix of the couplings, using the HRJSD method from (Figures 1A,C, 2A,C) IDC_LR_ and (Figures 1B,D, 2B,D) IDC_HR_ patients, respectively.

**Figure 3:**
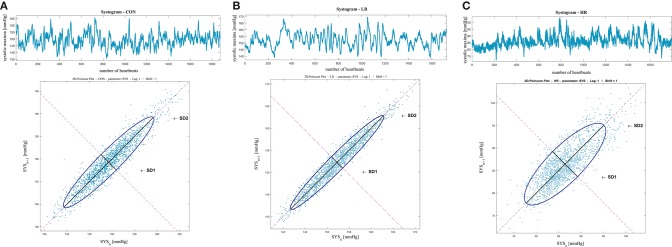
Systolic blood pressure Poincaré plot analysis results from **(A)** a CON subject, **(B)** a *IDC*_*LR*_ patient, and **(C)** a *IDC*_*HR*_ patient.

Figure 3 shows an example of the Poincaré plot method applied to a patient for each analyzed group, considering the systogram from (Figure 3A) a CON subject, (Figure 3B) a IDC_LR_ patient, and (Figure 3C) a IDC_HR_ patient.

**Figure 4:**
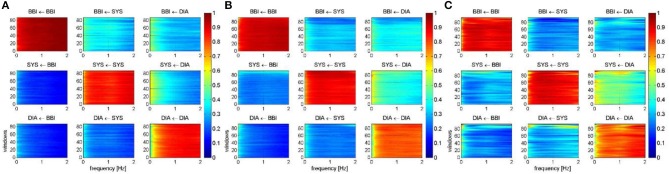
Averaged NSTPDC plots for cardiovascular coupling analyses for **(A)** the CON, **(B)** the *IDC*_*LR*_, and **(C)** the *IDC*_*HR*_ group. Arrows indicating the causal coupling direction from one time series to another time series, e.g., SYS←BBI, indicating the causal link from BBI to SYS. Coupling strength ranges from blue (0, no coupling) to red (1, maximum coupling) where BBI are beat-to-beat intervals, and SYS are successive end-systolic blood pressure amplitude values over time.

Figure 4 represents the averaged NSTPDC applied on the BBI, SBP, and DBP time series couplings, for (Figure 4A) the CON, (Figure 4B) IDC_LR_, and (Figure 4C) IDC_HR_ groups.

**Figure 5:**
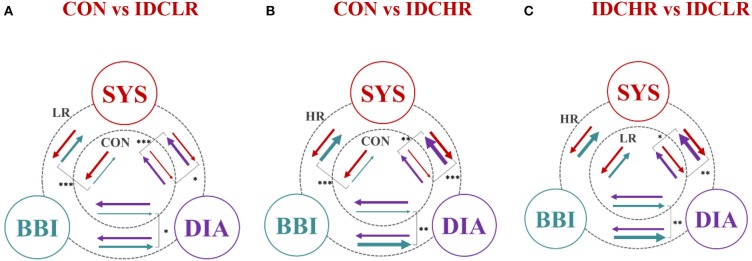
Graphical representation of the entire cardiovascular coupling structure (coupling strengths and directions) among the cardiac (BBI), systolic blood pressure (SYS), and diastolic blood pressure (DIA) systems, when comparing **(A)** CON vs. IDC_LR_, **(B)** CON vs. IDC_HR_, and **(C)** IDC_HR_ vs. IDC_LR_. The arrows direction indicates the causal coupling direction and the thickness the coupling strength. *Indicates that the NSTPDC index associated to the coupling represented by the arrow is statistically significant when *p ≤ 0.01; **p ≤ 0.001; ***p ≤ 0.0000167.

Figure 5 presents the relationships between all three analyzed groups, where the arrows represent the coupling direction and the arrow thickness indicating the coupling strength. In addition, the level of statistical significance has been represented (*p* ≤ 0.01).

When comparing the *IDC*_*HR*_ and *IDC*_*LR*_ groups, 96 indices presented statistically significant differences, corresponding to: 76 indices from the HRJSD, 12 from the SPPA, and 7 from the NSTPDC. After the correlation analysis, a total of 36 statistically significant indices were chosen for the classification process. Some of the most relevant indices, expressed in a mean value and 95% confidence interval, are shown in Table 4.

**Table 4:** Mean value and 95% confidence interval of the most significant indices when comparing the IDC_HR_ and IDC_LR_ patients.

**Index**	**IDC_**HR**_ (*N* = 14)**	**IDC_**LR**_ (*N* = 77)**	***p*-value**
PPAs_SD1/SD2	0.26 [0.22; 0.30]	0.20 [0.18; 0.22]	^*^
PPAd_SD1/SD2	0.27 [0.25; 0.28]	0.19 [0.17; 0.20]	^**^
PPAd_SD1	1.21 [1.15; 1.26]	0.92 [0.85; 0.98]	^**^
SPPAcd_Column_5	13.97 [13.54; 14.39]	12.09 [12.01; 13.02]	*
SPPAcd_Column_8	13.77 [13.53; 14.03]	12.71 [12.35; 13.06]	n.s.
HRJSDcd_E1-LD1	0.03 [0.024; 0.035]	0.09 [0.081; 0.091]	^**^
HRJSDcd_ LD1-V	0.056 [0.039; 0.073]	0.025 [0.021; 0.028]	^**^
HRJSDds_P-LU1	0.028 [0.022; 0.034]	0.016 [0.013; 0.019]	^**^
HRJSDds_V-LD1	0.050 [0.040; 0.060]	0.026 [0.022; 0.029]	^**^
NSTPDCcd_NF	−0.15 [−0.44; 0.14]	−0.71 [−0.85; −0.56]	*
NSTPDCcd_Area_c-d.	0.26 [0.23; 0.29]	0.20 [0.18; 0.21]	^**^

* ρ ≤ 0.01;

** *ρ ≤ 0.001; n.s. ρ ≥ 0.01; IDC_HR_, idiopathic cardiomyopathic patients at high risk of IDC_LR_, idiopathic cardiomyopathic patients at low risk of SCD; PPA, Poincaré plot analysis; SPPA, segmented Poincaré plot analysis; HRJSD, high resolution joint symbolic dynamic; NSTPDC, normalized short-time partial directed coherence; cd, cardio-diastolic coupling; ds, diastolic-systolic coupling*.

### IDC Patients vs. CON Subjects

When comparing IDC patients vs. CON subjects, 261 indices presented statistically significant differences between the two groups: 169 from the HRJSD, 76 from the SPPA, and 13 from NSTPDC. A total of 82 indices remained after discarding the highly correlated indices. Table 5 shows the most relevant of them.

**Table 5:** Mean value and 95% confidence interval of the most significant indices comparing IDC patients and CON subjects.

**Index**	**IDC (*N* = 91)**	**CON (*N* = 49)**	***p*-value**
HRJSDds-E0d	0.018 [0.014; 0.021]	0.0064 [0.0048; 0.0071]	^***^
NSTPDCcs_Area_c-s	0.25 [0.23; 0.28]	0.15 [0.13; 0.17]	^***^
PPAc_SD1	14.18 [10.44; 17.93]	23.27 [20.70; 25.84]	^**^
PPAc_SD1/SD2	0.29 [0.23; 0.34]	0.36 [0.33; 0.38]	^**^
HRJSDcd-E2d	0.02 [0.18; 0.21]	0.05 [0.045; 0.054]	^*^
HRJSDcd-LD1d	0.23 [0.22;0.24]	0.28 [0.27; 0.29]	^**^
HRJSDds_LD1-E2	0.0014 [0.0010; 0.0018]	0.0053 [0.0035; 0.0071]	^***^
HRJSDcd_E0-LA1	0.0004 [0.00028; 0.00053]	0.00014 [0.00010; 0.00019]	^***^
HRJSDds_LD1-LD1	0.069 [0.063; 0.074]	0.093 [0.086; 0.10]	^***^

* ρ ≤ 0.01;

** ρ ≤ 0.001;

*** *ρ ≤ 0.0000167; n.s. ρ ≥ 0.01; IDC, idiopathic cardiomyopathic patients; CON, control group; PPA, Poincaré plot analysis; HRJSD, high resolution joint symbolic dynamic; NSTPDC, normalized short-time partial directed coherence; cd, cardio-diastolic coupling; cs, cardio-systolic coupling; ds, diastolic-systolic coupling*.

### **IDC**_**HR**_ and **IDC**_**LR**_ Patients vs. CON Subjects

When comparing *IDC*_*HR*_ patients vs. CON subjects and *IDC*_*LR*_ patients vs. CON subjects, the differences were found in 182 and 247 indices, respectively. After the correlation analysis, 61 and 85 remaining indices were chosen for the classification step. A summary of the most relevant indices is shown in Tables 6, 7.

**Table 6:** Mean value and 95% confidence interval of the most significant indices comparing the IDC_HR_ patients and CON subjects.

**Index**	**IDC_**HR**_ (*N* = 14)**	**CON (*N* = 49)**	***p*-value**
HRJSDds_LD1-V	0.05 [0.040; 0.066]	0.02 [0.020; 0.027]	^***^
NSTPDCcs_Area_c->s	0.29 [0.24; 0.35]	0.15 [0.13; 0.17]	^***^
NSTPDCcd_Area_c->d	0.31 [0.27; 0.36]	0.22 [0.20; 0.24]	^**^
SPPAds_Column_2-6	27.23 [25.73; 28.72]	22.44 [21.26; 23.63]	^**^
HRJSDcd_LD1-E2	0.003 [0.001; 0.006]	0.012 [0.010; 0.015]	^**^

** ρ ≤ 0.001;

*** *ρ ≤ 0.0000167; IDC_HR_, idiopathic cardiomyopathic patients at high risk of sudden cardiac death; CON, control group; SPPA, segmented Poincaré plot analysis; HRJSD, high resolution joint symbolic dynamic; NSTPDC, normalized short-time partial directed coherence; cd, cardio-diastolic coupling; cs, cardio-systolic coupling; ds, diastolic-systolic coupling*.

**Table 7:** Mean value and 95% confidence interval of the most significant indices comparing the IDC_LR_ patients and CON subjects.

**Index**	**IDC_**LR**_ (*N* = 77)**	**CON (*N* = 49)**	***p*-value**
HRJSDcd-E1d	0.33 [0.29; 0.37]	0.21 [0.17; 0.26]	^**^
NSTPDCcs_Area_c-s	0.25 [0.22; 0.27]	0.15 [0.13; 0.17]	^***^
SPPAcd_Column1–8	5.40 [4.95; 5.85]	7.19 [6.56; 7.82]	^**^
HRJSDcd_E0-LA1	0.0004 [0.0002; 0.0005]	0.0014 [0.0010; 0.0019]	^***^
HRJSDds_LD1-LD1	0.070 [0.064; 0.075]	0.093 [0.086; 0.101]	^***^

** ρ ≤ 0.001;

*** *ρ ≤ 0.0000167; IDC_LR_, idiopathic cardiomyopathic patients at low risk of sudden cardiac death; CON, control group; SPPA, segmented Poincaré plot analysis; HRJSD, high resolution joint symbolic dynamic; NSTPDC, normalized short-time partial directed coherence; cd, cardio-diastolic coupling; cs, cardio-systolic coupling; ds, diastolic-systolic coupling*.

### HRV and BPV Standard Indices and DSM Results

When the HRV and BPV standard indices and the DSM were evaluated in the IDC_HR_ vs. IDC_LR_ comparison, no statistically significant indices were found. In the remaining comparisons, seven indices presented statistical significances: two from the sequence analysis and five from the HRV and BPV standard indices. A summary of these results is shown in Table 8.

**Table 8:** Mean value, 95% confidence interval and *p*-value of the HRV and BPV standard indices and the dual sequence method across all comparisons.

**Index**	**IDC_**HR**_ (*N* =14)**	**IDC_**LR**_ (*N* = 77)**	**CON (*N* = 49)**	***p*-value**	***p*-value**	***p*-value**	***p*-value**
				**IDC_**HR**_ vs. IDC_**LR**_**	**IDC vs. CON**	**IDC_**HR**_ vs. CON**	**IDC_**LR**_ vs. CON**
bslope	5.36 [3.49; 7.22]	7.81 [6.8; 8.75]	10.29 [9.09; 11.49]	n.s.	^**^	^**^	^*^
tslope	5.51 [3.75; 7.27]	7.94 [7.05; 8.83]	11.01 [9.78; 12.24]	n.s.	^**^	^**^	^**^
BBI_meanNN	828.42 [775.26; 881.57]	906.95 [879.27; 934.63]	883.79 [853.34; 914.23]	n.s	n.s	n.s.	n.s.
BBI_sdNN	33.44 [25.06; 41.81]	36.98 [32.8; 41.09]	47.82 [43.45; 52.19]	n.s.	^**^	^*^	^**^
BBI_rmssd	17.43 [12.00; 22.87]	20.53 [18.22; 22.84]	32.90 [28.25; 37.55]	n.s.	^**^	^*^	^**^
BBI_pNN50	2.48 [1.45; 3.52]	3.44 [2.73; 4.14]	0.14 [0.09; 0.18]	n.s.	^**^	^*^	^**^
BBI_HFn	0.65 [0.60; 0.70]	0.67 [0.63; 0.70]	0.62 [0.58; 0.67]	n.s.	n.s.	n.s.	n.s.
BBI_LFn	0.34 [0.29; 0.39]	0.32 [0.29; 0.36]	0.37 [0.32; 0.41]	n.s.	n.s.	n.s.	n.s.
BBI_LF/HF	2.48 [1.45; 3.52]	3.44 [2.73; 4.14]	2.55 [2.06; 3.04]	n.s.	n.s.	n.s.	n.s.
SYS_meanNN	121.3 [107.6; 135]	112.6 [108.3; 117]	122.5 [116.7; 128.2]	n.s.	n.s.	n.s.	^**^
DIA_meanNN	61.5 [55.2; 67.9]	58.5 [55.8; 61.2]	60.6 [56.7; 64.5]	n.s.	n.s.	n.s.	n.s.
DIA_VLF	0.48 [0.4;.0.56]	0.48 [0.45; 0.51]	0.39 [0.35; 0.44]	n.s.	^**^	n.s.	^**^

* p ≤ 0.01;

** *p ≤ 0.001; n.s. p ≥ 0.001; IDC_HR_, idiopathic cardiomyopathic patients at high risk of sudden cardiac death; IDC_LR_, idiopathic cardiomyopathic patients at low risk of sudden cardiac death; CON, control group; bslope, bradycardic fluctuations; tslope, tachycardic fluctuations; BBI, beat-to-beat cardiac interval; SYS, systolic blood pressure; DIA, diastolic blood pressure*.

### Classification Results

**Figure 6:**
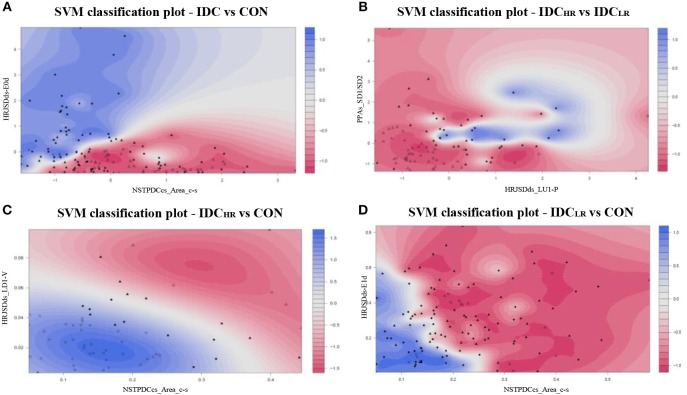
SVM classification plots: **(A)** IDC vs. CON (Laplace kernel), **(B)** IDC_HR_ vs. IDC_LR_ (Laplace kernel), **(C)** IDC_HR_ vs. CON (Gaussian kernel), and **(D)** IDC_LR_ vs. CON (Laplace kernel).

After the SVM classification step, the PPAs_SD1/SD2 and HRJSDds_LU1-P indices were deemed the optimal choices for the Laplace kernel SVM model, achieving an accuracy of 98.9% and an AUC of 0.96 for the *IDC*_*HR*_ vs. *IDC*_*LR*_ comparison. The HRJSDds-E0d and NSTPDCcs_Area_c-s allowed us to classify *IDC* patients from the CON group with an accuracy of 93.6 % and an AUC of 0.94 using the Laplace kernel. Meanwhile, the HRJSDds_LD1-V and the NSTPDCcs_Area_c-s were able to discriminate between *IDC*_*HR*_ patients and the CON group with an accuracy of 96.9% and AUC of 0.95 applying the Gaussian kernel. Finally, the HRJSDds-E1d and the NSTPDCcs_Area_c-s were found to be the best indices to classify *IDC*_*LR*_ patients from the CON group, obtaining 89.6 % accuracy and a 0.85 AUC with the Laplace kernel. The classification plots and the results are shown in Figure 6 and Table 9, respectively.

**Table 9:** Accuracy (Acc), sensitivity (Sn), specificity (Sp), and area under the curve (AUC) obtained with the best SVM model for each comparison.

	**IDC_**HR**_ vs. IDC_**LR**_**	**IDC vs. CON**	**IDC_**HR**_ vs. CON**	**IDC_**HR**_ vs. CON**
Indices	PPAs_SD1/SD2 HRJSDds_LU1-P	HRJSDds-E0d NSTPDCcs_Area_c-s	HRJSDds_LD1-V NSTPDCcs_Area_c-s	HRJSDds-E1d NSTPDCcs_Area_c-s
C	7	5.5	1	0.3
Kernel	Laplace	Laplace	Gaussian	Laplace
σ	1	1.5	0.3	1
Acc (%)	98.9	93.6	96.8	84.9
Sn (%)	100	93.7	92.9	89.6
Sp (%)	93.1	95.5	98.0	79.5
AUC	0.96	0.94	0.95	0.85

### Cascaded Risk Stratification

**Figure 7:**
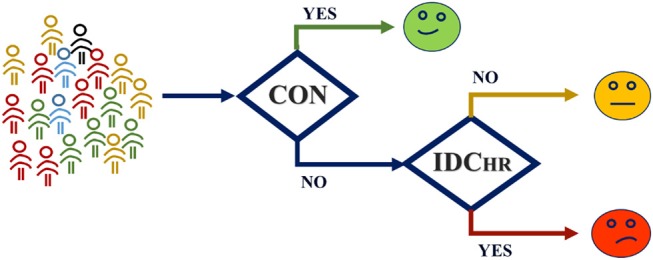
Cascade model structure. The subject is evaluated in the IDC vs. CON model, and resulting IDC patients' level of risk is evaluated by means of the HR vs. LR model.

In order to consider the typical clinical case in which the original condition of the subject is unknown, a cascade model was developed to classify this new subject using the label SDC risk, comparing the IDC vs. CON and IDC_HR_ vs. IDC_LR_ models (Figure 7). The general idea of this model is to classify the subject in either CON, LR, or HR without any prior labeling. The first step is to decide if a subject is an IDC patient or not using the IDC vs. CON model. Afterwards, those classified as IDC patients are analyzed based on the LR vs. HR model to predict their risk level.

The cascade model achieved 94.4% accuracy. From a One vs. All approach, the results for the CON group were 86.1% sensitivity and 92.8% specificity, from the perspective of the HR group, the model yielded a sensitivity of 93.2 and specificity of 94.5%, and for the LR group, the model achieved 98.8% sensitivity and 89.0% specificity.

## Discussion

The aim of this study was to find indices capable of stratifying SCD risk in IDC patients. In order to achieve this, we derived indices extracted from BBI (ECG) and systolic and diastolic blood pressure (BP) temporal series using linear and non-linear univariate and bivariate (coupling analysis) techniques. The indices extracted from these techniques revealed patterns that behave differently in patients at high risk of SCD. These indices (mainly from coupling analyses) were used to train several SVM models in order to classify the subjects based on different levels of SCD risk.

Our findings revealed that the cardio-diastolic coupling (NSTPDCcd_NF) is bidirectional with the diastolic activity as a driver in IDC_LR_ patients. Additionally, the coupling strength from cardiac activity over both the systolic and diastolic blood pressure (NSTPDCcs_Area_c->s, NSTPDCcs_Area_c->d) gets stronger when the patients are at high risk, suggesting that the cardiac activity is significantly dominating the blood pressure. This type of relationship has been observed before in congestive heart failure patients (Marinazzo et al., 2006).

Our results also revealed that there is a significant increase in systolic pressure activity as a response to the alternant activity of diastolic pressure in patients at risk of SCD (HRJSDds_P-LU1). Similar patterns were also observed in the cardio-diastolic coupling (HRJSDcd_LD1-P), suggesting that the deterioration of autonomic regulation is more severe in patients at high risk of SCD. Earlier studies indicated that symmetric patterns in the HRJSD could be related to baroreflex-like response patterns. This suggests that this kind of behavior is also more pronounced in patients at high risk (Baumert et al., 2005; Schulz et al., 2016).

The Poincaré plot analysis revealed that the patients from the HR group have higher short-term systolic blood pressure deviation than the patients from the LR group and the CON subjects. This pathological behavior is also reflected in the PPAs_SD1/SD2 index, which is less balanced as the illness progresses. The diastolic blood pressure behaved in a similar way: the short-term diastolic blood pressure deviation was significantly lower in HR patients and their PPAd_SD1/SD2 was less balanced as well, indicating higher BPV in patients with critical conditions. In earlier studies (Tatasciore et al., 2013; Ribeiro et al., 2017), higher short-term BPV was associated with several cardiac maladies such as left ventricular systolic dysfunction and atherosclerosis, amongst others. This BPV behavior was also present in sinoaortic denervated cats (Di Rienzo et al., 1991). Additionally, baroreflex effectiveness has been studied in paraplegic subjects (Castiglioni et al., 2007), and it their BPV, compared to control subjects, was found to be higher.

The aforementioned patterns were also present when the IDC patients were compared to the CON group. In general, the indices found in the PPA suggest that short-term BPV is higher in patients with pathological conditions (Mancia et al., 1983; Mehlum et al., 2018). The PPA indices regarding the BBI revealed that short-term deviation and the short- and long-term deviation ratio of the heart rate is higher in the CON group, suggesting that the HRV in the CON group is higher than in IDC patients. Additionally, the BBI_rmssd index suggests that the HRV is lower in IDC patients compared with the CON subjects (Shaffer and Ginsberg, 2017). It is known that reduced HRV is a predictor of an adverse prognosis in patients with cardiac disease (Task-Force-of-the-European-Society-of-Cardiology-the-North-American-Society-of-Pacing-Electrophysiology, 1996; Gang and Malik, 2003). Several studies have related low HRV with heart failure (Dekker et al., 2000; Musialik-Lydka et al., 2003; Sandercock and Brodie, 2006; Patel et al., 2017). Additionally, an increase complexity in BBI randomness and a lower fractal-like behavior have been associated with SDC (Huikuri et al., 2000).

The DSM revealed that both the tslope and bslope are significantly lower in IDC patients compared to CON subjects, and lower values of these sequences are associated with baroreflex dysfunction (Di Rienzo et al., 2001; La Rovere et al., 2008). In addition, a trend emerged in these indices when the patients were compared by their level of risk: IDC_HR_ patients showed lower tslopes and bslopes.

The segmented approach of the Poincaré Plot analysis revealed that some patterns in the cardiovascular coupling are more common in HR patients. There was a significantly higher concentration of these patterns in column 5 and 8 in both cardio-diastolic and cardio-systolic couplings in patients at higher risk of SDC, indicating a lower variability in their baroreflex activity compared with patients at low risk. SPPA patterns (SPPAcd_Column_5, SPPAcd_Column_8) occurred more frequently and were more concentrated in low risk patients, suggesting that these patients present a higher HRV compared to patients at higher risk of SCD. The viability of HRV as a reliable predictor of SCD in IDC patients was questioned in an earlier study (Grimm et al., 2003), which did not support the hypothesis that HRV is a reliable predictor of SCD in IDC patients. However, their results were based on a time domain analysis of HRV only, whereas the significant indices analyzed in our work come primarily from non-linear methods. These characterization methods are more suitable for describing the non-linear behavior of HRV in pathological conditions.

The HRJSD results suggest that patients at high risk adapt less frequently to changes in blood pressure, reflected in the lower presence of decreasing patterns of BBI (E0) in response to decreasing patterns in diastolic blood pressure (LD1). This may be a result of the vagal response, causing less frequent parasympathetic activity, leading to a less effective control of the blood pressure, and consequently the heart rate, in patients at higher risk conditions.

These results were consistent when CON subjects were compared with IDC patients: decreasing patterns (E0) in diastolic BPV were reflected in decreasing heart rates at higher frequencies in CON subjects. The DIA_LF/HF was higher in the patients when compared to the CON group. Higher levels of this index reflects efferent sympathetic activity (Mccraty and Shaffer, 2015). Additionally, a higher prevalence of unchanging patterns (E1) was found in the IDC patients compared with the CON group, indicating that changes in blood pressure are frequently not reflected in changes in heart rate in patients with pathological conditions.

In addition, the HRJSDcd-E2d and HRJSDcd-LD1d indices showed that steady (E2) and low decreasing (LD1) diastolic blood pressure patterns, independent from all BBI patterns, are more frequent in CON subjects. These indices suggest a worsened circulatory homeostasis in IDC patients and support the idea of the influence of baroreflex activity in pathological conditions (Kishi, 2018).

Earlier studies (Gavish et al., 2008; Schillaci and Pucci, 2010) have stated that the relationship between systolic and diastolic blood pressure should be coherent: if one of them increases, the other is expected to increase as well. The results of the systolic-diastolic coupling revealed that patterns that are opposing in nature (sLU1-dP, sLD1-dV) are more frequent in the HR group. This suggests that the relationship between systolic and diastolic blood pressure loses linearity as the pathological condition worsens.

The coupling strength of the cardiovascular and diastolic-systolic couplings are stronger in pathological conditions. In addition, the symmetric patterns of the diastolic-systolic coupling activity were less frequent in patients. This may be caused by the effect of the autonomic regulation mechanisms in pathological conditions (Floras, 2009; Kishi, 2012).

To summarize, our results suggest that there is a gradual loss of HRV as SCD risk increases and, at the same time, BPV increases alongside SCD risk. There is great controversy about the prognostic value of linear time and frequency domain HRV indices for risk stratification among this type of patient (Grimm et al., 2003; Hohnloser et al., 2003; Minamihaba et al., 2003; Valencia et al., 2009; Voss et al., 2010, 2012a). The results of this work support the idea that the commonly used techniques for analysis of the time and frequency domain of HRV is not suitable for risk stratification. However, the combination of non-linear HRV analysis and linear as well as non-linear coupling analysis seems to be a promising tool for risk assessment in IDC patients (Voss et al., 2012b; Valencia et al., 2013; Fischer and Voss, 2014). The processes involved in the circulatory homeostasis are by nature non-linear. Therefore, the differences between the stages of this process can be more adequately revealed by the quantification of the signal properties rather than by the assessment of their magnitude.

We hypothesize that a dysfunction of the vagal activity, and in general of the baroreflex mechanism as a whole, prevents the body from maintaining circulatory homeostasis correctly. This reduction in vagal activity and increase in the sympathetic influence exposes the cardiovascular system to frequent states of stress that contribute to the worsening of the condition over time. The gradual worsening of the heart rate and blood pressure variability's in the different SDC risk stages considered here supports this assumption. This kind of impairment has also been associated with other cardiac pathological conditions like ventricular fibrillation during myocardial ischemia (La Rovere et al., 2001). Abnormal sympathetic neural firing has been associated with SCD and the genesis of ventricular tachyarrhythmias (Esler et al., 1995). A similar behavior has been observed in elder mice (Freeling and Li, 2015), who presented a reduced baroreflex bradycardic response compared to younger mice.

This study has some limitations that are important to consider. The average age in the CON group was lower than in the IDC group, the influence of age in the study of HRV has been widely studied in earlier work (Voss et al., 2015). However, this limitation does not affect our results for HR vs. LR comparisons. A higher number of indices were analyzed than the number of patients in the database. Consequently, in order to minimize problems due to possible overfitting, we stablished different levels of statistical significance to reduce the dataset dimensionality, including the Bonferroni-Holm correction criterion.

The characterization of linear and non-linear coupling could be also analyzed based on linear and non-linear Granger causality in time- and frequency domains (Schulz et al., 2013a). The objective of this study was to evaluate the general behavior of the underlying coupling, through the average of all features (windows) over time was performed. Nevertheless, it is uncertain if another time-invariant time domain measure based on Granger causality would be more appropriate and would have more discriminative power (Porta and Faes, 2016). It would be interesting for an ongoing study.

Another limitation of this work is related to comorbidities and confounding factors influencing the autonomic regulation system. Therefore, these exclusion criterions make risk stratification not yet applicable to every patient.

## Conclusion

With this study we suggest that indices from coupling analysis and non-linear HRV and BPV can contribute to the development of risk stratification in IDC patients. We have introduced a novel cascade model that successfully classified subjects into different levels of SCD risk (CON, IDC_LR_, and IDC_HR_). Further, this study allowed us to uncover, for the first time, some of the complex interactions that take place within autonomic regulation, leading to a more accurate modeling, and interpretation of these processes in pathological conditions. Our findings revealed that there is a gradual decrease of HRV and an increase of BPV as the SCD risk increases. We conclude that patients at high risk of SCD can no longer maintain circulatory homeostasis consistently, leading to states of stress that worsens the condition over time.

However, these results should be validated with a greater number of patients, especially in the high-risk group. Therefore, the results presented in this work are more of a hypothesis-generating nature than confirmatory.

## Ethics Statement

This study are part of German Autonomic Regulation Trial (ART) study, oriented to evaluate risk predictors of SCD and to improve the risk stratification in IDC patients. Signals from 220 IDC patients were recorded in two hospitals: the Friedrich-Schiller University Hospital in Jena (44 patients) and the Franz-Volhard Clinic in Berlin (176 patients). Each study was performed following the recommendations of the Declaration of Helsinki and patient consent was granted. The study was approved by the local Ethics Committee (No. 0986-11/02).

## Author Contributions

BG and AV: conceptualization. JR, SS, BG, and AV: methodology, validation, writing—original draft preparation, writing—review and editing, and final version approval. BG, JR, and SS: software and data analysis. AV: data acquisition.

### Conflict of Interest Statement

The authors declare that the research was conducted in the absence of any commercial or financial relationships that could be construed as a potential conflict of interest.
